# Acupuncture Treatment of a Patient with Persistent Allergic Rhinitis Complicated by Rhinosinusitis and Asthma

**DOI:** 10.1093/ecam/nep240

**Published:** 2011-02-10

**Authors:** Ae-Ran Kim, Jun-Yong Choi, Jong-In Kim, So-Young Jung, Sun-Mi Choi

**Affiliations:** ^1^Acupuncture, Moxibustion and Meridian Research Center, Korea Institute of Oriental Medicine, Daejeon, Republic of Korea; ^2^Department of Acupuncture & Moxibustion, College of Korean Medicine, Kyung Hee University, Seoul, Republic of Korea; ^3^Division of Standard Research, Korea Institute of Oriental Medicine, Daejeon, 305-811, Republic of Korea

## Abstract

A pathophysiologic relationship between allergic rhinitis and rhinosinusitis and asthma has long been suggested. However, few clinical studies of acupuncture have been conducted on these comorbid conditions. A 48-year-old male suffering from persistent allergic rhinitis with comorbid chronic rhinosinusitis and asthma since the age of 18 years was studied. He complained of nasal obstruction, sneezing, cough, rhinorrhea and moderate dyspnea. He occasionally visited local ear-nose-throat clinics for his nasal symptoms, but gained only periodic symptom relief. The patient was treated with acupuncture, infrared radiation to the face and electro-acupuncture. Needles were inserted at bilateral LI20, GV23, LI4 and EX-1 sites with De-qi. Electro-acupuncture was performed simultaneously at both LI20 sites and additional traditional Korean acupuncture treatments were performed. Each session lasted for 10 min and the sessions were carried out twice a week for 5 weeks. The patient's Mini-Rhinoconjunctivitis Quality-of-Life Questionnaire score decreased from 38, at the beginning of treatment, to 23, 3 weeks after the last treatment. The Total Nasal Symptom Score was reduced from six (baseline) to five, 3 weeks after the last treatment. There was significant clinical improvement in the forced expiratory volume in 1 s—from 3.01 to 3.50 l—with discontinuation of the inhaled corticosteroid, and no asthma-related complaints were reported. Further clinical studies investigating the effectiveness of acupuncture for the patients suffering from allergic rhinitis and/or rhinosinusitis with comorbid asthma are needed.

## 1. Introduction

The co-existence of asthma with upper airway disorders such as rhinitis and rhinosinusitis has been recognized by physicians since Galen's era [1]. On this point, there is some evidence to support the concept that the upper and lower airways share several common anatomical and pathophysiologic features [2].

Patients with allergic rhinitis (AR) and concomitant asthma tend to have more severe symptoms, and uncontrolled AR or sinus diseases can aggravate asthma [3, 4]. Furthermore, the direct medical costs of one or more co-existing airway disease are greater than the direct costs of AR or asthma alone [2].

Despite the lack of vigorous and large, randomized controlled trials (RCTs) on acupuncture for the treatment of AR [5], several recent, small RCTs of acupuncture showed favorable efficacy in AR [6–8]. In addition, acupuncture is now widely administered for nasal or paranasal symptoms and practitioners of acupuncture have reported satisfactory efficacy in nasal and sinus symptoms [9]. However, acupuncture studies on upper and lower airway conditions, that is, AR and asthma, respectively, did not consider the comorbid status of the other section of the airway [3, 10]. Herein, we present a case of a male suffering from AR and concomitant rhinosinusitis and asthma.

## 2. The Case

### 2.1. Case History

A 48-year-old male suffering from nasal obstruction, sneezing, coughing, rhinorrhea and moderate dyspnea visited our clinic on August 12, 2008. He had suffered persistent nasal symptoms since the age of 18 years and additional diagnoses of bronchial asthma and hypertension had been made 3 years prior to his presentation at our clinic. He occasionally visited local ear-nose-throat (ENT) clinics for relief of his AR symptoms. In addition, his AR was aggravated by alcohol intake. During the spring season, he used intranasal corticosteroids about twice a week without satisfactory relief, and did not take any prescribed medications for AR, despite the presence of severe symptoms when he visited our clinic. For his asthma, he used dry powder inhalers (Budesonide 160 mg/formoterol 4.5 mg per dose, Symbicort Turbuhaler 160/4.5 *μ*g) irregularly about once to twice in every 3 days, as well as taking one tablet of theophylline (200 mg) and one tablet of montelukast sodium (10.4 mg) orally in the evenings. Additionally, he took one tablet of diltiazem (180 mg), one tablet of amlodipine (5 mg) and one capsule of aspirin (100 mg, enteric coated) once daily. There was no known family history of these conditions. At the baseline, before acupuncture treatment, the total serum immunoglobulin value was 183.8 IU ml^−1^ and the complete blood cell and routine serum biochemistry tests were normal.

The allergic skin prick test showed a positive reaction toward cat epithelium, where the allergen/histamine ratio was 1+. A chest X-ray showed an inactive lung lesion, and radiography of the paranasal sinuses was interpreted as pansinusitis and septal deviation without a nasal polyp.

During auscultation, wheezing was heard in both lung areas and was prominently associated with forced expiration. In the pulmonary function test (PFT), forced vital capacity (FVC) was 3.45 l (predicted value: 78%) and forced expiratory volume in 1 s (FEV1) was 3.01 l (predicted value: 83%).

### 2.2. Treatment

We obtained informed consent for our case study from the patient. Multiple treatments of acupuncture with electrical stimulation and infrared radiation therapy were performed twice per week, for 5 weeks, under the traditional medical diagnosis of stagnation of Qi and blood in nasal and paranasal area combined with lung deficiency. As for lung deficiency, we determined this diagnosis from his asthmatic symptoms accompanied by pale tongue and weak radial pulse. All acupuncture needles were disposable and the size was 0.3 mm in diameter and 40 mm in length (Dongbang Inc., Korea). The main local acupuncture was performed at the GV23, EX-1, bilateral LI20 and bilateral LI4 sites. For GV23 and EX-1, needles were inserted transversely toward the nose, to a depth of 0.2 cun. For bilateral LI20, needles were inserted obliquely toward their ipsilateral nostrils to a depth of 0.3 cun. For bilateral LI4, perpendicular insertion was performed to a depth of 0.5 cun.

The above-mentioned acupuncture points were manipulated by the lifting-thrusting, reinforcement-reduction and twirling reinforcement-reduction methods. The patient confirmed when De-qi was achieved.

Additional acupuncture was performed at the left LU9, SP3, LU10 and HT8 sites. For the left LU9, needles were inserted obliquely toward the distal direction, parallel to the left abductor pollicis longus tendon. For the left SP3, the needle was inserted obliquely toward the heel of the foot, parallel to the first left metatarsal bone. For the left LU10, the needle was inserted obliquely toward the wrist parallel to the first left metacarpal bone. For the left HT8, the needle was inserted obliquely toward the wrist and parallel to the borderline between the fourth and fifth left metacarpal bones. These treatment manipulations for four acupuncture points (LU9, SP3, LU10 and HT8) together are called Pye-jeonggeok, and are intended for the supplementation of lung meridian according to Sa-am acupuncture therapy [11]. Sa-am acupuncture therapy is a unique school of acupuncture in traditional Korean medicine initiated by Sa-am characterized by applying the five phases theory and mother—child supplementation and draining principle to the selection of points and needling manipulation [11, 12].

Electro-acupuncture was performed on both LI20 sites using a pulse stimulator (Ito Co., Japan) at 2 Hz frequency, 1 mA and 1 ms duration for 10 min; mild contraction of muscles around LI20 was observed. The face was exposed to infrared radiation (NET-1300, Megamedical, Korea) during an acupuncture needle insertion time of 10 min. All treatments were carried out by an acupuncture specialist who had been practicing clinically for nine years.

### 2.3. Case Outcome

Improvement in the AR symptoms was evaluated using the Total Nasal Symptom Score (TNSS) and the Mini-Rhinoconjunctivitis Quality-of-Life Questionnaire (MiniRQLQ). TNSS is a scoring system where patients mark one of the five severity choices (0 = none, 1 = mild, 2 = moderate, 3 = severe, 4 = very severe) for each of the four typical nasal symptoms (rhinorrhea, nasal obstruction, sneezing and nasal itching). The TNSS was calculated by summation of all four symptom scores. For each visit, the patient recorded his nasal symptoms for the TNSS scores at the clinic, and an additional diary was kept to evaluate nasal symptoms for the TNSS scores at home, during the week before the start of treatment and through all 5 weeks of acupuncture treatment.

The MiniRQLQ consists of five domains with a total of 14 questions: two questions for practical problems, and three questions each for activity limitations, nose, eye and other symptoms [13]. The MiniRQLQ is a self-administered scoring system. The patient marks one of the seven Likert-like scales (0 = no impairment to 6 = maximum impairment) for each of the 14 questions.

The TNSSs recorded at each visit to the clinic were six, four and five at baseline, termination points of acupuncture treatment at 5 weeks and 24 days after the last treatment session, respectively. As for the domains of TNSS, the symptom score of itching—two at baseline—reduced to zero from the second week of treatment to the end of the observation period. The MiniRQLQ score was 38 before the first acupuncture treatment. The score changed to 27 at the endpoint of 5 weeks of treatment and 7 days after the last treatment. At the last follow-up visit (24 days after the last treatment), the score decreased to 23 (Table 1).

At the last follow-up visit, no wheezing sounds were heard. The FEV1 increased from 3.01 l (predicted value: 83%) at baseline, to 3.50 l (predicted value: 99%) at the last observation. The FVC had also increased from 3.45 L (predicted value: 78%) to 3.53 l (predicted value: 81%) at this visit. In addition, the patient stopped using the oral inhaler completely after the first session of acupuncture treatment until the last follow-up visit without any deterioration of asthmatic symptoms.

From images of the paranasal sinus series, mild improvement was seen in both maxillary sinuses after treatment, compared with before the acupuncture treatment. Total serum IgE levels decreased from 183.8 IU ml^−1^ before treatment to 112.9 IU mml^−1^ by the end of the follow-up. There were no adverse events related to the acupuncture treatment. At the end of the follow-up, the patient stated that the acupuncture treatment was effective in relieving both his asthmatic and AR symptoms.

## 3. Discussion and Conclusion

In this case, the patient was initially diagnosed as Qi and blood stagnation in nasal and paranasal area from his nasal symptoms which were the chief complaints and lung deficiency from his asthmatic symptoms combined with his tongue and pulse signs [14]. Therefore, we planned to make a treatment protocol to disperse the stagnated Qi and blood in the nasal and paranasal area with acupuncture combined with electrical stimulation, infrared radiation therapy and the supplementing acupuncture method for the lung (LU9, SP3, LU10 and HT8).

As for TNSS changes seen on clinic visits, the TNSS of six just before the first treatment decreased to four after 5 weeks. The change of TNSS is not significant, as the baseline values were not so severe. Although the patient's nasal symptoms themselves were not clearly improved, the mean MiniRQLQ score, which is a more comprehensive outcome measurement for AR than TNSS, decreased from 2.71 to 1.93, and the difference of 0.78 was greater than the minimal clinically important difference (0.42) [15]. The improvement in MiniRQLQ was sustained until the last observation (Table 1).

The patient also suffered from asthma, which is known to have a close relationship with AR and rhinosinusitis. On his first visit, he was suffering from asthmatic symptoms in spite of conventional medical management for asthma. However, the PFT at the last observation showed an improvement from baseline. The extent of improvement in FEV1 was 490 ml; 16% of baseline FEV1 value, 3.01 l. This change probably represents clinically significant bronchodilation, according to international guidelines for the interpretation of pulmonary tests [16].

From these observations, it seemed that there were clinically significant improvements in the patient's quality-of-life regarding the upper airway conditions of AR and pansinusitis, and the pulmonary function regarding the lower airway condition of asthma, despite the lack of a causal link between the treatment and clinical improvement. Immunologically, there are some evidences that acupuncture can correct the imbalance between T-helper 1 cell-derived and T-helper 2 cell-derived pro-inflammatory and anti-inflammatory cytokines in allergic diseases [17]. The improvement of allergic conditions in our case might be partially due to immunomodulatory effect of acupuncture for both upper and lower airway allergic inflammations.

Systematic reviews on acupuncture treatment of AR and asthma showed no obvious efficacy but are limited by insufficiently well-designed RCTs [5, 10]. So far, clinical studies have been carried out under the notion that AR and asthma are discrete entities, in spite of the substantial co-existence of both upper and lower airway abnormalities.

**Figure 1:**
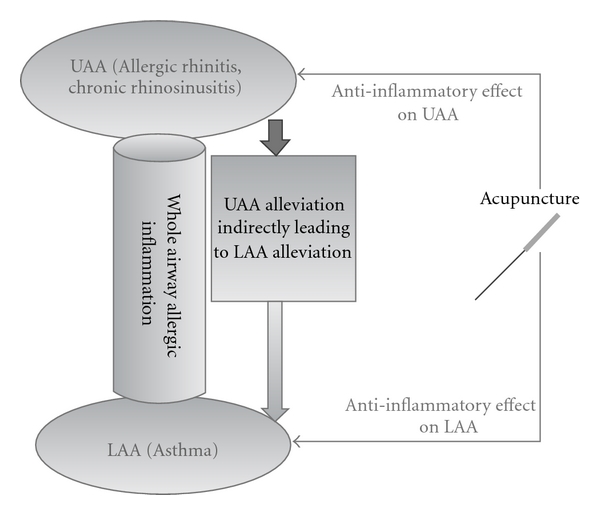
Possible mechanism of the effect of acupuncture on the whole airway allergic inflammation. UAA; upper airway allergy, LAA; lower airway allergy.

The airways of the nasal cavity and related paranasal sinuses belong to the respiratory tract, and the similarity and mutual complementarity between nasal and bronchial mucosa have been raised as an important issue [18]. In this context, the concept of “one airway, one disease” has been suggested, noting that AR and asthma are closely related [19]. Although a causal relationship between AR and asthma has not been established, there is some evidence that treatment for AR or rhinosinusitis can induce significant improvements in asthma [18, 20–22]. In one study that showed statistically significant improvement of bronchial hyperresponsiveness by intranasal corticosteroids for patients suffering from both AR and asthma, a simultaneously conducted radiolabeled examination showed that <2% of intranasally administered corticosteroids remained in the chest area [23]. This implies that the improvement of bronchial condition is not directly mediated by anti-inflammatory effect of intranasal corticosteroid on bronchus but indirectly caused by improved nasal function or reduced nasal inflammation. In our case, the improvement of lung function might be due to this indirect effect of the alleviation of allergic inflammation of nose or nasal function improvement as well as the anti-inflammatory effect of acupuncture such as the regulation of pro-inflammatory and anti-inflammatory cytokines related to asthma. A suggested direct and indirect mechanism of the effect of acupuncture on AR and/or chronic rhinosinusitis with comorbid asthma is depicted in Figure 1.

Further clinical research that considers patients with comorbid diseases of upper and lower airways (i.e., AR and/or rhinosinusitis with comorbid asthma) is needed in order to determine the clinical efficacy of acupuncture therapy. 

## Figures and Tables

**Table 1:** Changes in MiniRQLQ Scores.

Domain	Baseline	Primary endpoint (5 weeks after Baseline)	7 days after primary endpoint	24 days after primary endpoint
Activities				
Regular activities at home and at work	3	2	2	2
Recreational activities	3	2	2	2
Sleep	3	2	2	2
Practical problems				
Need to rub nose/eyes	2	1	1	1
Need to blow nose repeatedly	4	3	3	2
Nose symptoms				
Sneezing	3	2	2	2
Stuffy blocked nose	4	3	4	3
Runny nose	3	3	3	2
Eye symptoms				
Itchy eyes	2	1	1	1
Sore eyes	1	1	1	1
Watery eyes	2	1	1	1
Other symptoms				
Tiredness and/or fatigue	2	2	2	1
Thirst	2	2	1	1
Feeling irritable	4	2	2	2

Total score (mean score)	38 (2.71)	27 (1.93)	27 (1.93)	23 (1.64)
